# Editorial: Calcium signaling in cancer immunity

**DOI:** 10.3389/fimmu.2023.1315490

**Published:** 2023-10-30

**Authors:** Consuelo Amantini, Maria Beatrice Morelli

**Affiliations:** ^1^ School of Biosciences and Veterinary Medicine, University of Camerino, Camerino, Italy; ^2^ School of Pharmacy, Experimental Medicine Section, University of Camerino, Camerino, Italy

**Keywords:** cancer, cancer immunity, calcium signaling, lymphocytes, myeloid-derived suppressor cells, ion channel, tumor microenvironment

It is now widely recognized that Ca2+ represents an important and universal second messenger responsible for the regulation of various cellular processes such as proliferation, differentiation, migration and death (1). Moreover, a calcium signaling aberration has been identified as one of the parameters that contribute to tumor development and progression in an intricate and heterogeneous network. While multi-omic pan-cancer studies have clarified and improved our understanding of cancer molecular biology by highlighting several oncogenic drivers and cancer hallmarks (2, 3), understanding how calcium concentrations are regulated in tumor cells remains an interesting challenge. In fact, studies have demonstrated that, on the one hand, the dysregulation of intracellular Ca2+ levels is involved in tumor initiation and progression, and on the other hand, Ca2+ signaling modulates the tumor microenvironment through proliferation, apoptosis, and the immune infiltration (4). These multiple roles make it impossible to determine, with precision, whether the dysfunction of calcium signals is the cause of a tumor or is the result of other oncogenic changes. Hence, further research needs to be conducted on Ca2+ pumps, Ca2+-dependent kinases, exchangers, and channels, including voltage-gated, CRAC, Orai, STIM, MUC, and TRP, in order to inhibit tumor development and enhance anti-cancer immunity.

To date, several findings highlighted the role of cytosolic Ca2+ signals, regulated by channels, in stimulating proliferation and maturation of CD8+ lymphocytes and natural killer cells (5), in promoting immune cell migration and chemotaxis (6) and in endorsing the formation of immune synapse and cell target killing (7). In agreement, Sala et al. demonstrated that the modulation of the Ca2+ levels mediated by the ether a-gò-gò related gene 1 (ERG1) influences selection and differentiation pathways of lymphoid clones. In particular, the authors underlined the importance of ERG1 activity to sustain an adequate electrochemical gradient necessary to achieve the Ca2+ influx during B and T cell receptor activation. Dysregulation of ERG1 leads to alterations in Ca2+ signals that allow the wrong selection of proliferating tumorigenic lymphoid clones. In line with these results, it has been demonstrated the aberrant expression of ERG1 is found in leukemia and it is associated with chemoresistance and worse prognosis (8). The importance of Ca2+ levels for the survival of T effector lymphocytes is also highlighted by Yang et al. who described the essential role of the Ca2+ import into the mitochondria, induced by intrinsic levels of dimethylguanidino valeric acid, in the survival of T effector lymphocytes during the expansion.

There is no doubt that the knowledge of how calcium can influence the survival and activation of lymphocytes opens new prospects for enhancing the immune response against tumors. However, attention must also be paid to the tumor microenvironment capable of inducing immunosuppression and therefore nullifying the effectiveness of the immune infiltrate with serious limits in the efficacy of cancer immunotherapy. In fact, the tumor microenvironment is characterized by the secretion of soluble factors and cytokines that promote the conversion of myeloid cells into myeloid-derived suppressor cells (MDSCs) (9). High concentrations of MDSCs, immature heterogeneous cells derived from hematopoietic stem cells, are associated with cancer development and consequently poor prognosis. Their immunosuppressive role is mainly to inhibit the antigen-specific CD8+ T-cell function by inducing cell cycle arrest and apoptosis. Thus, the elimination of MDSCs by reducing their differentiation, expansion, activation or immunosuppressive ability, represents an ambitious aim in clinical therapies. Clearly, to achieve this fundamental objective, it is necessary to understand in detail what signals control MDSC formation and accumulation in tumor microenvironment. In this regard, in breast cancer, Huang et al. demonstrated that blocking Ca2+/calmodulin-dependent protein kinase 2 (CAMKK2) reduces tumor growth through CD8+ stimulation (10). CAMKK2 is a serine/threonine kinase activated by Ca2+-triggered signaling cascade that is overexpressed in several cancers such as hepatocellular carcinoma and gastric cancer (11). Huang et al. also used CAMKK2-/- MDSCs in *in vitro* experiments. They demonstrated that deletion of CAMKK2 enhances apoptotic cell death and stimulates terminal differentiation of MDSCs by promoting accumulation of reactive oxygen species. They also determined that syngenic lymphoma is not able to survive in animals knock down for the CAMKK2 gene. The deletion of CAMKK2 leads to a markedly reduced accumulation of MDSCs in the tumoral stroma with consequent increased T cell response. These results identify CAMKK2 as new therapeutic target to restrict MDSCs activity and improve the anti-cancer immunotherapy.

A lot of attention has been focused on ion channels in cancer recently, especially those that modulate Ca2+ flux. In fact, their aberrant expression and dysfunction, in both cancer and anti-tumor immune cells, affect immune escape mechanisms and, consequentially, tumor progression. About this, Han et al. identified for head and neck squamous cell carcinoma (HNSCC) a 12-ion-channel-gene signature useful to predict the prognosis. Among these channels, ANO1 is a calcium-activated chloride channel whose overexpression promotes distant metastasis of HNSCC whereas TRPC1 is a cation channel that mediates Ca2+ influx and found to be associated with tumorigenesis. The 12-ion-channel-gene signature also correlates with the immune infiltration: in the low-risk group the levels of B lymphocytes and CD8+ T cells are high while the high-risk group is characterized by the presence of resting-state macrophages seem to be associated with a negative prognosis.

Despite many calcium-mediated pathways involved in tumor progression and antitumor immunity being investigated, integrating the findings obtained so far with those derived from new and sophisticated proteomic analyses will be the new challenge. Omics-based strategies, fundamental to biomedical and translational research, present a chance to discover new pharmacological targets in cancer immunotherapy (12) by identifying all Ca2+-dependent players involved in the host-tumor relationship.

## Author contributions

CA: Writing – original draft. MM: Writing – review & editing.
